# Autoantibodies Targeting AT1 Receptor from Patients with Acute Coronary Syndrome Upregulate Proinflammatory Cytokines Expression in Endothelial Cells Involving NF-*κ*B Pathway

**DOI:** 10.1155/2014/342693

**Published:** 2014-11-30

**Authors:** Weijuan Li, Zhi Li, Yaoqi Chen, Songhai Li, Yuanyuan Lv, Wenping Zhou, Mengyang Liao, Feng Zhu, Zihua Zhou, Xiang Cheng, Qiutang Zeng, Yuhua Liao, Yumiao Wei

**Affiliations:** Laboratory of Cardiovascular Immunology, Institute of Cardiology, Union Hospital, Tongji Medical College, Huazhong University of Science & Technology, Wuhan, Hubei 430022, China

## Abstract

Our study intended to prove whether agonistic autoantibodies to angiotensin II type 1 receptor (AT1-AAs) exist in patients with coronary heart disease (CHD) and affect the human endothelial cell (HEC) by upregulating proinflammatory cytokines expression involved in NF-*κ*B pathway. Antibodies were determined by chronotropic responses of cultured neonatal rat cardiomyocytes coupled with receptor-specific antagonists (valsartan and AT1-EC2) as described previously. Interleukin-6 (IL-6), vascular cell adhesion molecule-1 (VCAM-1), and monocyte chemotactic protein-1 (MCP-1) expression were improved at both mRNA and protein levels in HEC, while NF-*κ*B in the DNA level was improved detected by electrophoretic mobility shift assays (EMSA). These improvements could be inhibited by specific AT1 receptor blocker valsartan, NF-*κ*B blocker pyrrolidine dithiocarbamate (PDTC), and specific short peptides from the second extracellular loop of AT1 receptor. These results suggested that AT1-AAs, via the AT1 receptor, induce expression of proinflammatory cytokines involved in the activation of NF-*κ*B. AT1-AAs may play a great role in the pathogenesis of the acute coronary syndrome by mediating vascular inflammatory effects involved in the NF-*κ*B pathway.

## 1. Introduction

Autoantibodies against the angiotensin II type 1 receptor (AT1-AAs) have been found in malignant and refractory hypertension, preeclampsia, and renal-allograft rejection patients in many studies [1–5]. Wallukat et al. [3] and Dragun et al. [5] found AT1-AAs activating the AT1 receptor have an agonist-like effect. In their study, the antibodies of AT1 receptor were detected by the chronotropic responses to AT1 receptor-mediated stimulation of cultured neonatal rat cardiomyocytes with receptor-specific antagonists. Studies [6, 7] in our lab found that the AT1-AAs can increase Ca^2+^ activation and Dechend et al. [8] and Griendling et al. [9] had demonstrated the function of activating the nicotinamide adenine dinucleotide phosphate (NADPH) oxidase and nuclear factor-*κ*B (NF-*κ*B) pathway in vascular smooth muscle cells [5]. And NF-*κ*B pathway was one of the main inflammation pathway.

So these studies suggested that AT1-AAs may play a role in inflammation-related disease, such as atherosclerosis. Atherosclerosis is a chronic inflammatory disease [10, 11], while whether AT1-AAs exist in patients with atherosclerosis and associated with the pathogenesis of atherosclerosis is little investigated. Angiotensin II, via the AT1 receptor, plays a critical role in the pathogenesis of atherosclerosis by inducing vascular inflammation [12, 13]. Angiotensin II modulates the expression of proinflammatory cytokines such as IL-6, MCP-1, and VCAM-1 in human endothelium [14], which stimulates recruitment and infiltration of mononuclear leukocytes into the vessel media at the initial stage. All in all, angiotensin II can increase atherosclerotic inflammation by activating the NF-*κ*B pathway [15], while AT1-AAs induce inflammation maybe via the same way to angiotensin II. In order to illuminate the role of AT1-AAs in the pathogenesis of atherosclerosis, our study investigated whether AT1-AAs can be found in patients with coronary heart disease and whether the antibodies can affect the related signaling pathways, mainly the NF-*κ*B pathway, and induce vascular inflammation.

## 2. Materials and Methods

### 2.1. Patients

We enrolled 64 coronary disease candidates who received coronary angiography at our hospital from September 2010 to October 2010. All the patients provided written informed consent. The study was approved by the Ethics Committee of Tongji Medical College. Patients were excluded with severe heart failure (left ventricular ejection fraction <30%), renal function failure (creatinine >3 mg/dL), or other manifested autoimmunologic disease or infectious disease. According to clinical history, laboratory examinations, and the coronary angiography results [16], we divided the patients into four groups, (1) acute coronary syndrome (ACS) group: the patients had unstable angina (without typical ECG and without cardiac troponin increase), non-ST elevated infarction (without typical ECG but cardiac troponin increase), and ST elevated infarction (typical ECG and cardiac troponin increase). (2) Stable coronary disease (SCD) group: the patients had a typical angina history and had a stenosis at least 50% in one or more main coronary. (3) Noncoronary disease (NCD) group: the patients had some coronary heart disease risk including diabetes, hypertension, and hyperlipidemia without related coronary disease evidence. (4) Control group: the candidates had normal coronaries and had no other heart diseases or heart risk factors diagnosed. Blood was collected during the course of ACS and the stable coronary disease, while the blood of noncoronary patients and control group was collected on admission. For serum preparation, the samples were centrifuged at 4,000 g for 30 min and stored at −20°C as described previously [3]. The C-reactive protein (CRP) levels were assayed by the Kriptor ultrasensitive immunofluorescent assay (Brahms, Germany), with a detection limit of 0.06 mg/L as described previously [17].

### 2.2. AT1-EC2 Peptide Synthesis

The AT1 receptor structure is a seven-transmembrane bundle intercalated by three extracellular loops (EC1, EC2, and EC3) and three intracellular loops and the EC2 is a key fraction for the extracellular signal sensation. Studies have indicated that agonistic antibodies are mainly directed at the EC2 loop [18]. According to our previous studies [2, 6, 7], the peptides corresponding to the second extracellular loop of the human AT1 receptor (AT1-EC2), whose amino acid sequences are IHRNVFFIENTNITVCAFHYESQNSTL (165aa–191aa), were synthesized by peptide synthesizer (PSSM-8, Shimadzu, Japan). These peptides were evaluated by high-performance liquid chromatography (HPLC) analysis on a Vydac C-18 column and 95% purity was achieved.

### 2.3. Assay of AT1-AAs and Isolation and Purification of the Antibodies from the Patients' Serum

**Figure 1:**
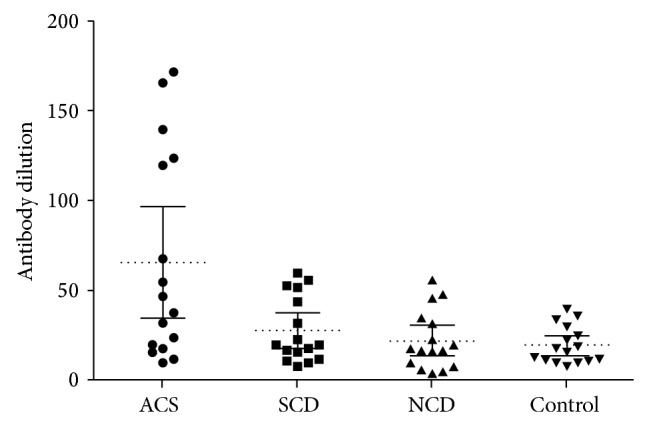
The titers of AT1-AAs in the sera sample collected from each group of patients with CHD measured by repeated ELISA. Titers of AT1-AAs in different group of patients and the healthy control group were all shown in the figure. The positive patient was defined as the titer higher than 1 : 40. AT1-AAs in ACS group were significantly different from the SCD, NCD, and control groups (*P* < 0.001) and the positive rate was 50%, 31%, 19%, and 6%. The AT1-AAs used for the following experiments were collected from the 5 positive sera samples of ACS group separately, with the antibody titer higher than 1 : 120.

Antibodies against AT1 receptor in the patients were detected by ELISA method as described previously Figure 1 [2, 3]. The antibody titers were measured three times with the sample collected in 3 different days and each sample was measured in three different plates. Briefly, AT1-EC2 peptide 10 *μ*g/mL in buffer was coated on microtiter plates. The wells were saturated with phosphate-buffer saline supplemented with 3% skimmed milk, 0.1% Tween 20, and 0.001 merthiolate. 50 *μ*L diluted sera from 1 : 40 to 1 : 320 were added to the coated plates overnight at 4°C. After additional washings, horseradish peroxidase-conjugated anti-mice IgG antibodies were added for 1 h at 37°C. The plates were then washed and the substrate was added for color display. Optical density was measured at 450 nm and the titer of antibody was calculated. The AT1-AAs were isolated and purified from the high titer (1 : 120) positive patients' sera with acute coronary syndrome, as described before [19]. In brief, the immunoglobulin fraction was isolated from serum samples by ammonium sulfate precipitation at a saturation of 50%, 40%, and 33%, three times. After centrifugation, the precipitates were dissolved in 2 mL PBS (0.01 mol/L, pH 7.4) and dialyzed at 4°C. The buffer was replaced several times during dialysis. The AT1-AAs IgG was prepared from the sera of 5 ACS patients with higher AT1-AAs titer separately, purified by affinity chromatography as described previously [11]. Briefly, the immunoglobulin fractions were loaded on a Sepharose 4B CNBr-activated gel, to which the peptide corresponding to AT1-EC2 was covalently linked [20]. The antibodies were adsorbed on the affinity column and eluted with 3 mol/L potassium thiocyanate. The concentration was assayed by ultraviolet visible spectrophotometry. IgG from ACS patients after AT1-AAs had been eluted was used as negative control and was termed “nonspecific” IgG [21].

### 2.4. Chronotropic Test on Cultured Neonatal Beating Cardiomyocytes

The neonatal heart cells were harvested from the minced ventricles of Wistar rat (1–3 days old) and prepared with the method of trypsin digestion [22]. They were grown in Dulbecco's Modified Eagles Medium (DMEM) (Gibco, USA) containing 10% fetal bovine serum (FBS). On the third day or fourth day, the cells were incubated for 2 h in 2 mL fresh serum-containing medium. Seven to ten selected cells or synchronously contracting cell clusters per flask were counted for 15 s on a heated desk (37°C) of an inverted microscope. This procedure was repeated twice in different cultures to yield results representing a total of up to 30 cells for each sample (referent to [3]). The basal contraction rate of the spontaneously beating cardiomyocytes was 150 ± 5/min. The increased beating rate was calculated after adding 20 *μ*g/mL (medium) purified AT1-AAs, or 0.1 *μ*M angiotensin II with or without 1 *μ*M AT1 receptor blocker valsartan or 1 *μ*g/mL AT1-EC2 peptide.

### 2.5. Cell Culture and Treatment

The human umbilical vein cell line, EA.hy926, was obtained from American Type Culture Collection (ATCC, USA) and was maintained in DMEM containing 10% FBS. The 70% confluent cells in culture flasks or six-well plates were preincubated for 24 hours in DMEM containing 0.2% bovine serum albumin and then treated with AT1-AAs (20 *μ*g/mL medium), angiotensin II (0.1 *μ*M medium), or other drugs. Cells were grown in a humidified atmosphere containing 5% CO_2_ at 37.0°C.

### 2.6. Real-Time Quantitative Polymerase Chain Reaction (RT-PCR)

Total RNA was extracted from the endothelial cells using RNAiso Plus reagent according to the protocol provided by the manufacturer (Takara Biotechnology, Japan). One microgram of total RNA was reverse transcribed using PrimerScript RT reagent Kit (Takara Biotechnology, Japan) and the resulting cDNA was used as a PCR template. The mRNA levels were determined by real-time PCR with ABI PRISM 7900 Sequence Detector system (Applied Biosystem, USA). SYBR green I (Takara Biotechnology, Japan) was used as the fluorescence indicator. GAPDH was used as endogenous control. Relative gene expression level was calculated using the comparative Ct method formula 2^−ΔΔCT^ [23]. Sequences of primers are as follows: using 5′-ATGAACTCCTTCTCCACAAGCGC-3′ and 5′-GTCAGGTCGGACTCCCGAGAAG-3′ for human IL-6; 5′-CCGGATTGCTGCTCAGATTGGA-3′ and 5′-AGCGTGGAATTGGTCCCCTCA-3′ for human VCAM-1; 5′-TGCAGAGGCTCGCGAGCTA-3′ and 5′-CAGGTGGTCCATGGAATCCTGA-3′ for human MCP-1; 5′-CCCTTCATTGACCTCAACTACATGG-3′ and 5′-AGTCTTCTGGGTGGCAGTGATGG-3′.

### 2.7. Western Blot

Western blot was performed as described previously [24]. Briefly, after treatment, the cells were lysed with lysis buffer (PH 7.2; 50 mM Tris base, 1 mM EDTA*·*2Na, 150 mM NaCl, 0.1% sodium dodecyl sulfate, 1% NP-40, 0.5% sodium deoxycholate, 0.1% phenylmethanesulfonyl fluoride) and total proteins were isolated by point vibration and centrifugation. Cytosolic protein and nucleoprotein were isolated by nuclear protein lysate containing protease inhibitors and phosphatase inhibitor. Protein concentration was determined by a BCA protein assay (Pierce, USA). Samples containing 40 *μ*g of total protein were loaded onto 7.5% to 15% SDS-polyacrylamide gel and transferred to nitrocellulose filter membrane (Bio-Rad, Hercules, CA, USA). After the blocking of nonspecific binding, proteins on the blots were detected with rabbit polyclonal antibodies to IL-6 (Epitomics, USA), VCAM-1 (Epitomics, USA), MCP-1 (Epitomics, USA), and NF-*κ*B p65 (Cell Signaling Technology, USA) and mouse polyclonal antibodies to I*κ*B*α* (Cell Signaling Technology, USA) and p-I*κ*B*α* (Cell Signaling Technology, USA), followed by the horseradish peroxidase-conjugated secondary antibody. Then, the proteins were visualized using a chemiluminescence method and quantified by using Quantity One V4.1 software and NIH Image J Version 1. 61. To verify equal protein loading and accuracy of results, GAPDH, *β*-actin (for total or cytosolic proteins), and histone-H1 (for nuclear proteins) were used as internal references.

### 2.8. Electrophoretic Mobility Shift Assays (EMSA)

As described previously [25], cells were stimulated with AT1-AAs (20 *μ*g/mL) for 15 min, 30 min, and 60 min. Nuclear extracts were prepared by point vibration and centrifugation. DNA binding activity was determined in (10–15 *μ*g) nuclear extracts from treated cells by binding with labeled NF-*κ*B consensus. Samples were applied to wells with 6% nondenaturing polyacrylamide gel. The general procedure was performed according to the instructions from the Chemiluminescent Nucleic Acid Detection Module Kit and Lightshift Chemiluminescent EMSA Kit (Pierce, USA).

### 2.9. AT1 Blocker, NF-*κ*B Blocker, and Short Peptides Blocking Effect Assay

To confirm the role of AT1-AA inducing NF-*κ*B and further increasing IL-6, VCAM-1 expression via AT1 receptor, we used Western blot to test the inhibiting effects of relative blockers such as valsartan for the AT1 receptor and PDTC for NF-*κ*B. In order to investigate the possible epitope of the second loop of AT1 receptor, we synthesized 5 overlapping short peptides as the interventional blocker in the IL-6 and MCP-1 expression. These were also tested by Western blot. The short overlapping peptides sequences were VFFIEN, ENTNIT, ITVCAF, AFHYESQ, and QNSTLPI.

### 2.10. Statistical Analysis

Results were expressed as mean ± SEM and significant differences among groups were assessed by one-way ANOVA, followed by a Duncan's multiple comparison posttest using SigmaStat 3.5 software (Systat Software, IL). The *χ* ^2^ test was used to access the significance of ratio and percentage data. Differences were considered statistically significant at a value of *P* < 0.05.

## 3. Results

### 3.1. The Presence of AT1-AAs in Patients

Clinical characteristics, biochemical parameters, and measures of CRP and AT1-AA were summarized in Table 1. There were no significant differences in common parameters, except the blood pressure in noncoronary patients group and left ventricular ejection fraction in acute coronary syndrome group which were related to the heart disease characteristics. The positive rate of IgG type AT1-AAs was 50%, 31%, 19%, and 6%, respectively, in the four groups. The mean AT1-AAs titer was 1 : 124, 1 : 53, 1 : 50, and 1 : 40 in positive patients (antibody titer > 1 : 40). The positive rate of AT1-AAs in acute coronary syndrome was significantly higher compared to control group (*P* < 0.001). Though the stable coronary disease group and noncoronary patients group had a higher positive rate, the statistics were not significant. The CRP level in the four groups was 5.6 mg/L, 4.7 mg/L, 3.0 mg/L, and 2.5 mg/L, respectively, and compared to control group, the acute coronary syndrome was significantly higher.

### 3.2. AT1-AAs Increase Neonatal Cardiomyocyte Beat Rate

**Figure 2:**
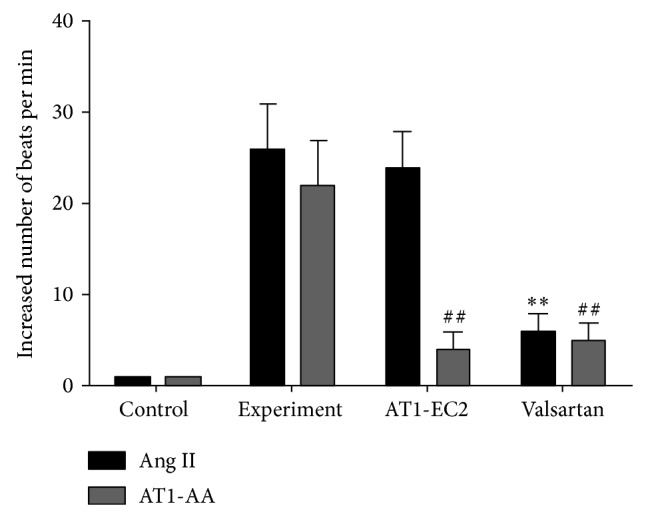
Chronotropic effects of angiotensin II or purified AT1-AAs from patients with acute coronary syndrome on cultured neonatal rat cardiomyocytes. Like angiotensin II (0.1 *μ*M), AT1-AAs (20 *μ*g/mL) increased cells beats about 22/min, and AT1 receptor blocker valsartan (1 *μ*M) and overload AT1-EC2 peptide (1 *μ*g/mL) could significantly inhibit this chronotropic effect demonstrating that AT1-AAs have an agonistic effect on the AT1 receptor. The nonspecific IgG (nsIgG) (20 *μ*g/mL) was used as control; the beats were not increased as is shown. Ang II: angiotensin II, AT1-AAs: autoantibodies against the AT1 receptor; ^**^ *P* < 0.01 versus Ang II control group; ^##^ *P* < 0.01 versus AT1-AAs control group. The data were organized from 5 independent experiments each treated with one of the positive samples and repeated three times.

We used the spontaneously beating rat neonatal cardiomyocyte to test the AT1-AAs activity. From Figure 2, we could find the purified IgG type AT1-AAs (20 *μ*g/mL) from 5 patients separately with acute coronary syndrome increased the beat rate 22 ± 5/min, compared with the control (exposed to the nonspecific IgG (nsIgG) from the same patients), and this effect could be abolished by both AT1 receptor blocker valsartan (1 *μ*M) and overdose AT1-EC2 peptide (1 *μ*g/mL), meaning AT1-AAs had an agonistic effects on AT1 receptor. The 0.1 *μ*M angiotensin II treatment had a similar effect.

### 3.3. AT1-AAs Induce IL-6, VCAM-1, and MCP-1 Expression

**Figure 3:**
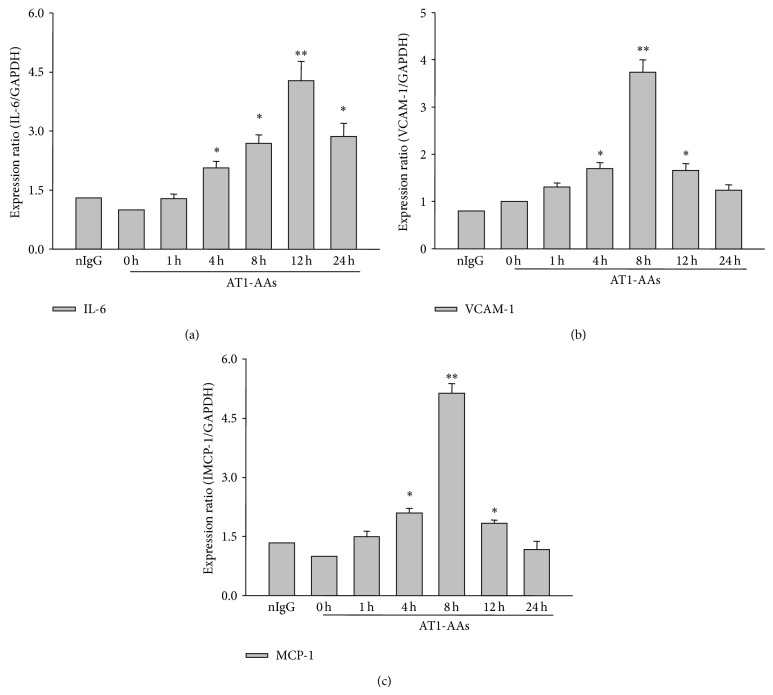
IL-6, VCAM-1, and MCP-1 mRNA expression in human endothelial cells stimulated by AT1-AAs. In cells incubated with AT1-AAs (20 *μ*g/mL) for 0, 1, 4, 8, 12, and 24 hours or human nonspecific IgG (20 *μ*g/mL) from the patients for 8 hours as a negative control, the IL-6 (panel (a)), VCAM-1 (panel (b)), and MCP-1 (panel (c)) mRNA levels were detected by real-time quantitative PCR analysis. *y*-axis represents the relative mRNA levels; ^**^ *P* < 0.01; ^*^ *P* < 0.05 versus 0 hour (control); *N* = 6. (nonspecific IgG group *n* = 1).

**Figure 4:**
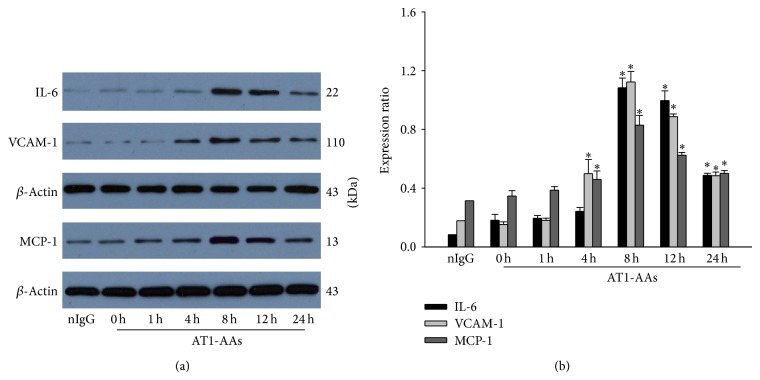
IL-6, VCAM-1, and MCP-1 protein expression in human endothelial cells stimulated by AT1-AAs. After incubation with AT1-AAs for 0, 1, 4, 8, 12, and 24 hours or human nonspecific IgG from the patients for 12 hours as a negative control, the IL-6, VCAM-1 and MCP-1 (panel (a)) protein levels were determined by Western blot analysis. *y*-axis represents the relative protein levels, *β*-actin used as a protein loading control. Panel (b) was the summarized data from 5 independent experiments; ^*^ *P* < 0.05 versus 0 hour (control); *N* = 6. (nonspecific IgG group *n* = 1).

The RT-PCR and Western blot analysis showed that AT1-AAs stimulated IL-6, VCAM-1, and MCP-1 mRNA and protein expression in endothelial cells significantly. The mRNA and protein expression levels were shown in Figures 3 and 4. In Figure 3, panels (a), (b), and (c) display IL-6, VCAM-1, and MCP-1 mRNA levels, respectively. In Figure 4, panel (a) is a typical Western blot band, and panel (b) is the summarized data from the 6 independent experiments (next figures like this model, including the typical figure and summarized data, have an experiment number equal to 6). As shown in Figures 3 and 4, the production of IL-6, VCAM-1, and MCP-1 mRNA and protein induced by AT1-AAs increased significantly in a time-dependent manner. Protein expression of IL-6 induced by AT1-AAs reached a maximal level at around 12 hours and then decreased gradually but continued to be higher than control for up to 24 hours. Similar to IL-6, VCAM-1 and MCP-1 mRNA and protein expression also increased in a time-dependent manner. However, the difference between IL-6, VCAM-1, and MCP-1 was that the latter reached the maximum expression levels at around 8 hours. The RT-PCR and Western blot assays addressed that AT1-AAs can induce endothelial cells to produce proinflammatory cytokines, which perhaps contribute to the pathogenesis of vascular inflammation.

### 3.4. AT1-AAs Induce IL-6 and VCAM-1 Expression via AT1 Receptor

**Figure 5:**
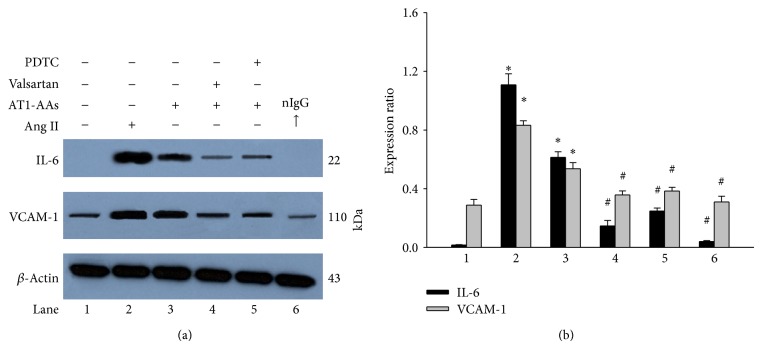
Effects of valsartan and PDTC on AT1-AAs or angiotensin II-induced protein expression in human endothelial cells. Valsartan (AT1 receptor blocker) and PDTC (NF-*κ*B inhibitor) were added to cell medium 2 hours before AT1-AAs treatment for 12 hours. Panel (a) shows the typical Western blot results. Panel (b) shows the summarized data (*n* = 5); ^*^ *P* < 0.05 versus control group (lane 1); ^#^ *P* < 0.05 versus AT1-AAs group (lane 3).

To test whether AT1-AAs regulate endothelial IL-6 and VCAM-1 expression via AT1 receptor, we observed the effect of AT1 receptor blocker valsartan on the AT1-AAs-induced protein expression. Cells were preincubated with valsartan for 2 hours and then treated with AT1-EC2-AA for 12 hours. As shown in Figure 5, AT1-AAs could increase IL-6 and VCAM-1 expression, but there was no difference between nonspecific IgG group and blank control group. Furthermore, valsartan not only abolished AT1-AAs-induced IL-6 secretion, but also inhibited the expression of VCAM-1 mediated by AT1-AAs.

### 3.5. AT1-AAs Activate NF-*κ*B Pathway

**Figure 6:**
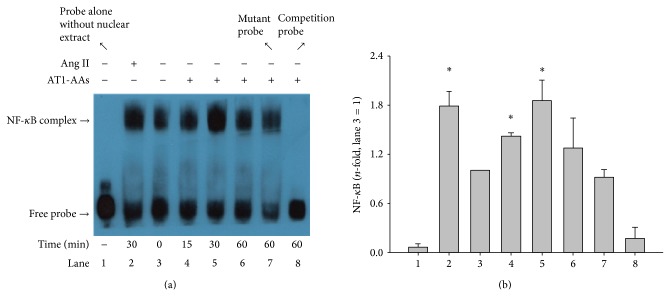
Activation of NF-*κ*B by EMSA analysis after AT1-AAs stimulation in endothelial cells. After incubating cells with angiotensin II (0.1 *μ*M) for 30 min and AT1-AAs (0.1 *μ*M) for 0, 15, 30, and 60 min, activity of NF-*κ*B in endothelial cells was determined by EMSA analysis (panel (a)). Lane 1: probe alone without nuclear extract; lane 2: incubation with angiotensin II for 30 min; lane 3: control (before incubation with AT1-AAs); lane 4: incubation with AT1-AAs for 15 min; lane 5: incubation with AT1-AAs for 30 min; lane 6: incubation with AT1-AAs for 60 min; lane 7: mutant oligonucleotide to test the specificity of the method; lane 8: 200-fold molar excess of unlabeled NF-*κ*B oligonucleotide as a competitive inhibitor. Data were summarized from 5 independent experiments (panel (b)); ^*^ *P* < 0.05 versus control group (lane 3).

To evaluate the possible mechanism of AT1-AAs-mediated IL-6 and VCAM-1 expression, EMSA was conducted to determine whether AT1-AAs were able to activate NF-*κ*B DNA binding activity. The unstimulated cells showed lower activation of NF-*κ*B. As shown in Figure 6, the NF-*κ*B activity of the treated cells with AT1-AAs increased as early as 15 min and reached a maximal effect at 30 min. Although these effects declined, the levels continued to be higher than control up to 60 min. The NF-*κ*B complex was competitively blocked with a 200-fold molar excess of unlabeled NF-*κ*B oligonucleotide; however, a mutant oligonucleotide showed no competition, indicating that AT1-AAs-induced NF-*κ*B complex was specific for the NF-*κ*B element.

### 3.6. AT1-AAs Induce Expression of p65

**Figure 7:**
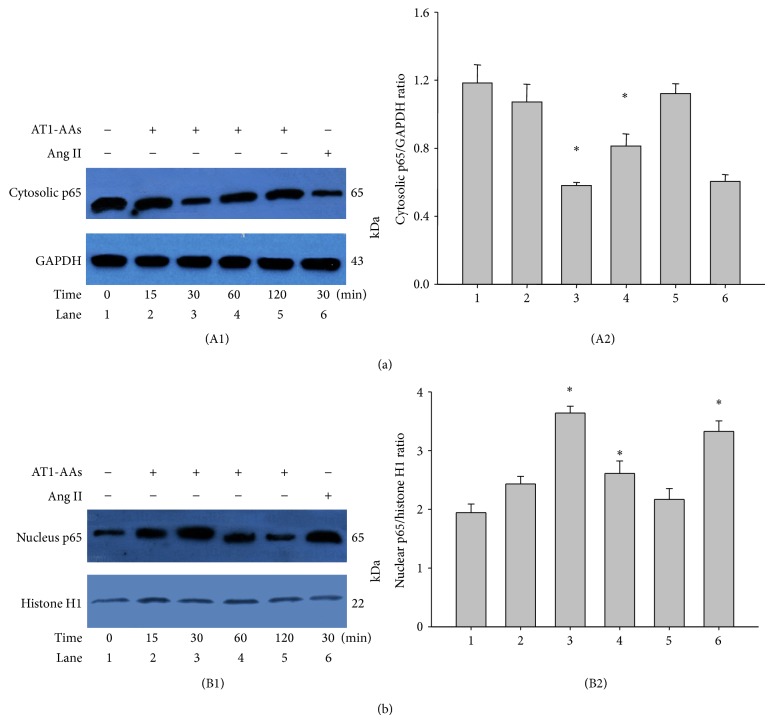
NF-*κ*B p65 expression in human endothelial cells stimulated by AT1-AAs. After incubation with AT1-AAs (0.1 *μ*M) for 0, 15, 30, 60, and 120 min and angiotensin II (0.1 *μ*M) for 30 min, p65 levels were determined by Western blot analysis. Panel (a): cytoplasm of p65 levels; panel (b): nucleus of p65 levels. Angiotensin II was used as positive control; ^*^ *P* < 0.05 versus 0 min (control) group; *N* = 5.

To examine the related component of NF-*κ*B activation, we observed nuclear translocation of p65 NF-*κ*B. We analyzed p65 protein levels in cytosolic (Figure 7(a)(A1)) and nuclear (Figure 7(b)(B1)) fractions after stimulation with AT1-AAs with different lengths of time. This data is summarized in Figures 7(a)(A2) and 7(b)(B2). From the figure, the p65 NF-*κ*B was mainly located in the cytoplasm before stimulation; after being stimulated with AT1-AAs, p65 gradually translocated to the nucleus. The maximal effect was manifested at around 30 min; then the effect gradually declined.

### 3.7. AT1-AAs Degrade Cytosolic Inhibitor *κ*B*α* (I*κ*B*α*) through Phosphorylation

**Figure 8:**
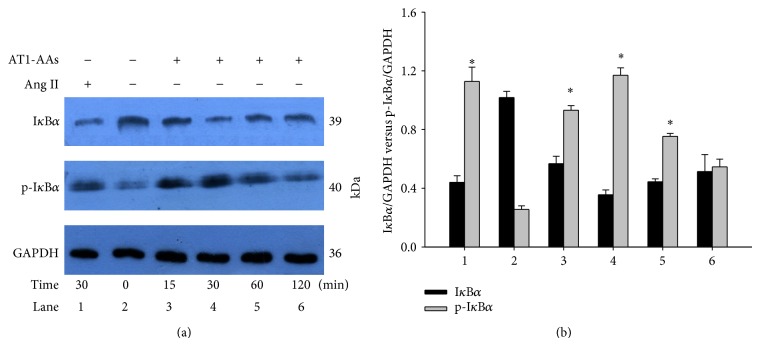
Cytoplasm I*κ*B*α* and p-I*κ*B*α* expression in human endothelial cells after stimulation with AT1-AAs. The cells were incubated with AT1-AAs after 0, 15, 30, 60, and 120 min and angiotensin II (0.1 *μ*M) for 30 min; I*κ*B*α* and p-I*κ*B*α* levels were determined by Western blot analysis (panel (a)). Angiotensin II was used as positive control (lane 1). Data were summarized in panel (b) from 5 independent experiments; ^*^ *P* < 0.05 versus 0 min (control) group (lane 2) in p-I*κ*B*α* group.

To further examine how AT1-AAs induce NF-*κ*B activation, we investigated whether AT1-AAs affect the phosphorylation and the subsequent degradation of I*κ*B*α* by Western blotting with antibodies against phosphor-specific I*κ*B*α* and I*κ*B*α* (Figure 8). I*κ*B*α* levels were reduced in AT1-AAs-stimulated cells up to 30 min and then returned to the basal level after 2 hours. Accordingly, p-I*κ*B*α* levels reached the maximal value after 30 min of AT1-AAs stimulation and slowly declined thereafter. These observations indicate that AT1-AAs cause rapid phosphorylation and degradation of I*κ*B*α*, followed by complete resynthesis in 2 hours. These effects were closely correlated with the time-course of AT1-AAs-induced NF-*κ*B activation and p65 nucleus translocation.

### 3.8. Effect of PDTC on AT1-AAs-Induced IL-6 and VCAM-1 Protein Expression

To examine whether activation of NF-*κ*B is related to IL-6 and VCAM-1 expression, we observed the effect of PDTC, an NF-*κ*B inhibitor, on the expression of IL-6 and VCAM-1 induced by AT1-AAs with Western blot. As shown in Figure 5, PDTC markedly attenuated IL-6 (from 0.614 ± 0.0378 to 0.248 ± 0.021; *P* < 0.05) and VCAM-1 (0.636 ± 0.042 to 0.414 ± 0.025; *P* < 0.05) protein expression compared with AT1-AAs group. This indicated the AT1-AAs, mainly via the NF-*κ*B pathway, regulate the IL-6 and VCAM-1 expression.

### 3.9. Effects of Short Peptides on AT1-AAs-Induced IL-6 and VCAM-1 Protein Expression

**Figure 9:**
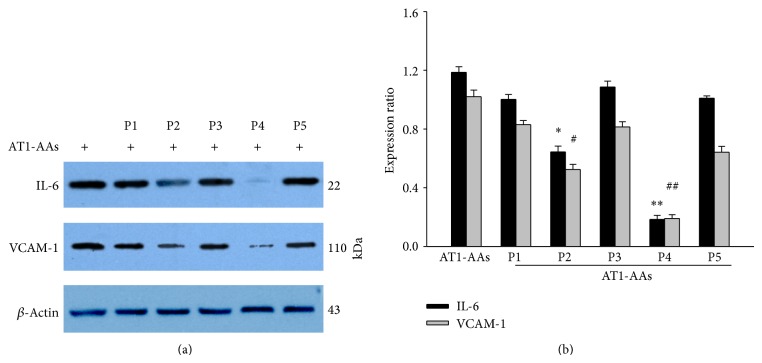
Effects of five short peptides on AT1-AAs-induced IL-6 and VCAM-1 expression in endothelial cells. The short peptides were added to cells medium 2 hours before AT1-AAs treatment for 12 hours. And the IL-6 and VCAM-1 expression were determined by Western blot (panel (a)). The five short peptides were P1, VFFIEN; P2, ENTNIT; P3, ITVCAF; P4, AFHYESQ; P5, QNSTLPI. Data were summarized from 5 independent experiments (panel (b)); ^*^ *P* < 0.05 versus AT1-AAs (control) group, IL-6 expression; ^#^ *P* < 0.05 versus AT1-AAs (control) group, VCAM-1 expression.

To investigate the key antigen epitope of the second loop of the AT1 receptor, we tested whether the overdose of short peptides from the second loop of AT1 receptor can block the AT1-AAs agonistic effects. As shown in Figure 9, we found that P4 peptide “AFHYESQ” and P2 “ENTNIT” markedly suppressed AT1-AAs-activated IL-6 expression compared with control group. The remaining peptides had no effect on AT1-AAs-activated IL-6 expression, even with an overdose. Similar findings had been observed in the study of AT1-AAs-induced VCAM-1 expression, further demonstrating that AT1-AAs, mainly via AFHYESQ and/or ENTNIT peptide fraction epitope, activate the AT1 receptor.

## 4. Discussion

We demonstrate the high titer of IgG type AT1-AAs in the patients with coronary heart disease especially in the patients with acute coronary syndrome, compared with patients without coronary disease. The purified AT1-AAs also increased the beats of rat neonatal cardiomyocytes, like angiotensin II. This study suggested that AT1-AAs exist in coronary atherosclerosis disease, especially with acute coronary syndrome. Jin et al. [26] have proved that the increased beats of neonatal cardiomyocytes could be induced by the IL-6. And there might be other factors affecting the beat rate. Our present study proved that AT1-AAs induce proinflammatory cytokines such as IL-6, VCAM-1, and MCP-1 expression in endothelial cells and activate NF-*κ*B pathway. This effect of AT1-AAs on NF-*κ*B-proinflammatory cytokines was mediated, at least partly, through the activation of AT1 receptor, since the valsartan and AT1-EC2 inhibited the beating increase (in Figure 2). This indicated that AT1-AAs had an agonistic effect similar to angiotensin II. The main mechanism of this agonistic effect perhaps involved the molecular mimicry theory, where AT1-AAs specifically bind to the second loop of AT1 receptor and activate the AT1 receptor. A typical example of an agonistic antibody causing disease is Graves' disease, in which the thyroid stimulating autoantibodies can activate the thyrotropin receptor [27]. Now, there is evidence indicating some cardiovascular disease was correlated with related autoantibodies. Fu et al. [28–30] have reported that anti-alpha adrenoreceptor may cause hypertension and Jahns et al. [31, 32] proved anti-beta-adrenoreceptor may cause dilated cardiomyopathy or Chagas heart disease through the agonistic mechanism and immunoglobulin absorption can improve the heart function in the antibody-induced cardiomyopathy [33–35]. The present study showed that AT1-AAs activity and its effects were also agonistic, which can be blocked not only by the specific AT1-EC2 peptide or short peptides with the relative epitope, but also by the AT1 receptor blocker valsartan.

There is abundant data, including our studies, which show atherosclerosis involves a series of inflammatory phagocytes, lymphocytes, proinflammatory cytokines, chemokines, adhesive molecules, related immunological or inflammatory receptors, and signaling pathways such as NF-*κ*B in the vascular wall [36–39]. NF-*κ*B is a transcription factor that regulates many genes involved in inflammation and the immune response. In this process, the initial lesion is mainly due to the endothelial cells dysfunction, which firstly activated and expressed a series of adhesion molecules and cytokines such as VCAM-1, ICAM-1, MCP-1, and IL-6, which promote the monocytes recruitment and the inflammatory reaction [10, 11, 40]. IL-6 is an important inflammatory regulator that mediates the migration and differentiation of macrophages and is a mediator of the hepatic acute-phase response. Our study has proved that AT1-AAs increased the expression of IL-6, VCAM-1, and MCP-1 (Figures 3 and 4), and this result indicated that the NF-*κ*B pathway was active during the development process of coronary atherosclerosis disease. Heart Outcomes Prevention Evaluation (HOPE) study [41] and Sata and Fukuda [42] have demonstrated that angiotensin II enhances the progression of atherosclerosis mainly through the NF-*κ*B pathway. In endothelial cells, angiotensin II binds to the AT1 receptor, to mediate a series of signaling cascades which then activates NF-*κ*B to initiate gene transcription [43]. NF-*κ*B includes five units, p50, p65 (RelA), c-Rel, p52, and RelB, and usually exists in the cytoplasm in the form of p65/p50 heterodimers. Generally, the heterodimers associate with inhibitory molecule I*κ*B*α* in an inactive form [44]. Angiotensin II can stimulate nuclear translocation of p65 subunit, DNA binding, transcription of an NF-*κ*B reporter gene, and I*κ*B degradation [45]. The present study observed that AT1-AAs degraded the I*κ*B*α* (Figure 8) by phosphorylation and the cytosolic and nucleus p65 expression was stimulated by AT1-AAs (Figure 9). EMSA results also demonstrated that AT1-AAs-stimulated endothelial cells can activate NF-*κ*B (Figure 6).

Our study collected autoantibodies from the acute coronary syndrome patients due to the high titer and the hypothesis that unstable coronary plaque rupture might be the main mechanism of ACS (according to the reference [46]) and the inducer of AT1-AAs. Evidence indicates that angiotensin II and IL-6 play a role in the pathogenesis of acute coronary syndrome [47]. Angiotensin II can stimulate IL-6 expression and colocalization with several inflammatory cytokines in the shoulder region of coronary plaques, the zone most prone to rupture. Blocking angiotensin production is one of the therapeutic goals of plaque stabilization, rendering it less susceptible to rupture and thrombosis [48]. While how AT1-AAs were induced is still unknown, Stepan et al. once studied the relationship of the infection of B19 virus and the production of AT1-AAs [49]. Subsequently, the AT1-AAs, through agonist activity targeting AT1 receptor, increase vascular inflammation, like in renal-allocation rejection [5]. This mimicry mechanism of AT1-AAs on the AT1 receptor and the initial production of AT1-AAs both need further exploration. AT1-AA may be one of the causes of ACS; a strategy of removing the autoantibody might be a step in that direction.

## Figures and Tables

**Table 1:** Main clinical features of enrolled patients and levels of CRP and AT1-AAs.

Characteristic	Acute coronary syndrome group	Stable coronary disease group	Noncoronary patients group	Control group
Patient number *n*	16	16	16	16
Male *n* (%)	10 (62.5)	9 (56.2)	8 (50)	9 (56.2)
Female *n* (%)	6 (37.5)	7 (43.8)	8 (50)	7 (43.8)
Age y	54 ± 12	63 ± 17	64 ± 12	56 ± 14
Body mass kg	69 ± 12	68 ± 6	66 ± 8	63 ± 10
Smoker *n* (%)	5 (31.2)	6 (37.5)	4 (25%)	5 (31.2)
Systolic blood pressure mmHg	135 ± 14	137 ± 7	148 ± 17^*^	129 ± 4
Plasma fasting glycerin mg/dL	112 ± 17	119 ± 25	121 ± 34	109 ± 9
LDL-cholesterol mg/dL	132 ± 18	126 ± 22	134 ± 23	122 ± 21
Left ventricular eject fraction %	49 ± 10^∗#^	55 ± 7	57 ± 8	59 ± 5
Serum creatinine mg/dL	1.3 ± 0.4	1.3 ± 0.2	1.4 ± 0.2	1.1 ± 0.2
Coronary disease history^&^ y	5 ± 4	9 ± 4	—	—
Multicoronary lesion^&^, *n* (%)	7 (44)	12 (75)	—	—
CRP level mg/L	5.6 ± 1.76^∗#^	4.7 ± 0.52	3.0 ± 1.11	2.5 ± 1.05
Positive AT1-AAs *n* (%)	8 (50)^∗#^	5 (31)	3 (19)	1 (6)

Mean titers of positive serum^&^	1 : 124	1 : 53	1 : 50	1 : 40

Statistics signs

^*^Compared to control group, *P* < 0.05.

^#^Compared to noncoronary patients group, *P* < 0.05.

^&^No statistics performed.
